# Fatigue in Egyptian patients with rheumatic diseases: a qualitative study

**DOI:** 10.1186/s12955-015-0304-7

**Published:** 2015-08-22

**Authors:** Mohamed Mortada, Amal Abdul-Sattar, Laure Gossec

**Affiliations:** Rheumatology & Rehabilitation Department, Zagazig University, Faculty of Medicine, 28 Qawmeia street, Zagazig, Egypt; Department of rheumatology, Sorbonne Universités, UPMC Univ Paris 06, Institut Pierre Louis d’Epidémiologie et de Santé Publique; AP-HP, Pitié Salpêtrière Hospital, F-75013 Paris, France

**Keywords:** Rheumatoid arthritis, Fibromyalgia, Fatigue, Patient perspective, Quality of life, Qualitative study

## Abstract

**Objectives:**

Fatigue is frequent in rheumatic diseases. Fatigue expression and consequences may be modified by cultural differences. Our objective was to increase the understanding of the fatigue experience and characteristics among Egyptian, Muslim patients with rheumatic diseases.

**Methods:**

Prospective monocentric qualitative study based on conventional qualitative content analysis, inductive reasoning, grounded theory. Egyptian patients with rheumatoid arthritis (RA), fibromyalgia or axial spondyloarthritis (AxSpA) were asked about fatigue, its patterns, consequences and self-management.

**Results:**

Of the 60 patients interviewed, 20 patients had each disease (RA, fibromyalgia and AxSpA); median ages ranged from 34 to 40 years. Patients were mainly male (*N* = 40, 66 %), had 3 to 7 years (mean) of disease duration and had moderate disease activity. Some aspects of the patients’ experience of fatigue may be specific to the Egyptian and Muslim culture such as the description of fatigue as a physical more than a mental impact of the disease, the response to the effect of fatigue on sexual function and the gender specific (women more than men) limitation of social activities due to fatigue which was more obvious in our study than other previous studies. Other aspects of patients’ experience of fatigue like overlap between the patients’ perception of fatigue and pain and coping strategies were similar to the findings in previous studies.

**Conclusion:**

This study gives insights regarding fatigue in rheumatic diseases in an Arabic and Muslim culture. Similarities and differences with previous studies were noted and should be taken into account when assessing these patients.

## Introduction

Fatigue is common across all rheumatic and musculoskeletal diseases (RMDs), with significant fatigue reported by 41–80 of patients with rheumatoid arthritis (RA) [1, 2]. The prevalence of fatigue has been reported to range from 40 % to 76 % in patients with osteoarthritis and fibromyalgia respectively [1] and from 60 to 74 % for those with axial spondyloarthritis (AxSpA) [3, 4].

Fatigue can have a substantial impact on patients’ self-care activities and overall quality of life [5–8]. It is often identified as one of the most challenging aspects of chronic rheumatic diseases [9–11]. The causes of fatigue appear multi-factorial in RMDs; disease activity plays a role but additional factors such as psychological distress and treatments may be additional causes of fatigue [12]. Pharmacological interventions for rheumatic diseases have a limited effect on fatigue [11, 13]; therefore, patients rely on self-management techniques.

There is an increasing trend to study the impact of fatigue in RMDs [14]: several quantitative studies have explored the causality of fatigue or its relationship with rheumatic disease activity. While qualitative studies are less frequent [11, 15–20], such studies may help in further exploration of the perceived causes, consequences and management of fatigue from the patients’ point of view by developing and understanding patients’ experiences of living with and managing fatigue [20]. This may allow health professionals to orient patients towards self-management strategies.

Cultural aspects may play a role in the expression of fatigue. Fatigue in RMDs has to date, to our knowledge, only been explored in Western-culture countries [15–24]. However, qualitative studies on fatigue have shown the importance of culture and religion. Indeed, patients’ beliefs and perceptions about diseases and pharmaceutical measures are important to explain coping strategies and these beliefs can be related to cultural background [25]. A qualitative study including patients with knee and hip osteoarthritis in the Dominican Republic, demonstrated that among this population beliefs about etiology included illness being caused by God’s will and exposure to water (e.g. bathing). Among these patients coping strategies reflected their beliefs about disease causation and included prayer, avoidance of water and giving a great importance to drainage of knee effusions [21]. Kumar et al, when performing interviews with RA and lupus patients of South Asian origin in the United Kingdom in 2011, found that patients who think that disease is ‘God’s will’ usually don’t believe that they can play an active role in managing their disease. In some cases, this attitude may hinder their compliance with treatment recommended by their doctor [22]. It also appeared these same patients believed that drugs in general were more overused and more harmful than did their Caucasian counterparts [23].

Thus, culture and religion are important aspects to consider when assessing disease impact. A recent multinational study (the COMORA study) has shown fatigue levels were variable across countries and these differences might be explained by inter-country differences rather than disease activity levels [25].

The current qualitative study aimed to increase the understanding of the fatigue experience and characteristics among non-Western culture, Egyptian and Muslim patients with rheumatic diseases: RA, fibromyalgia and AxSpA.

## Patients and methods

### Design

A prospective monocentric qualitative study was conducted by individual interviews. Ethical approval was obtained from the Institutional Review Board (IRB) at Zagazig university hospitals and all participants provided their informed consent before participation.

### Population

Outpatients visiting the ambulatory clinic in the Department of Rheumatology & Rehabilitation in Zagazig University (Zagazig, Egypt) were considered for this study. Three distinct groups of patients were invited to participate in this study, according to their diagnosis: (1) RA patients, fulfilling the 2010 American College of Rheumatology/European League Against Rheumatism (ACR /EULAR) classification criteria for RA [26]; (2) fibromyalgia patients, fulfilling the 2010 ACR preliminary diagnostic criteria for fibromyalgia [27]; and (3) AxSpA patients, fulfilling the ASAS criteria for classification of AxSpA [28]. Furthermore all patients had definite and established disease, according to their treating rheumatologist.

All patients were Egyptian and of Muslim religion, and were willing and able to come in for a thirty minutes interview one- on-on-one. Exclusion criteria included heavy handicap with impossibility to come to the hospital, language limitations (not speaking Arabic), and lack of informed consent. No patients were excluded based on age.

The final number of patients was determined based on previous indications that a sample size above 30 allows appropriate assessments in qualitative studies [29].

### General data collection

Eligible patients completed a questionnaire with the help of a nurse or a doctor if needed (given high illiteracy levels in the population). Questions concerned age, gender, marital status, work status, patient global assessment numeric rating scale, NRS from 0 to 10), current treatment, and fatigue assessment using a fatigue NRS (0–10) for fatigue severity and for fatigue consequences [30].

One of the two rheumatologists of the study (MM, AAS) filled in a global assessment of disease (0–10 NRS) and a disease activity scale specific to each group was assessed, as appropriate: Disease Activity Score on 28 joints (DAS28) for RA [31], Bath Ankylosing Spondylitis Disease Activity Index (BASDAI) for AxSpA [32] or Fibromyalgia Impact Questionnaire (FIQ) for fibromyalgia [33].

### Interviews

In this study, the individual interview method [34] was used based on conventional qualitative content analysis, inductive reasoning, and grounded theory [35].

A semi-structured interviewer guide was developed to guide the content of the interview and consisted of general and specific questions. The participants’ experiences, opinions and concerns relating to particular topics (Table 1) were explored, particularly centered on fatigue, its patterns and consequences, and self-management strategies pertaining to this fatigue.

**Table 1:** Main topics and items of discussion during interviews

Main topics	Items of discussion
Pattern of fatigue	Daily pattern
	Seasonal pattern
	Description of fatigue
	Impact of disease activity on fatigue
	Impact of sleep on fatigue
Consequences of fatigue	Effects on physical activity
	Effects on work
	Effect on leisure activity
	Effect on social activity
	Effect on child care
	Effect on relationships
Management	Coping strategies
	Potential help and support

### Data interpretation

Interviews were recorded by audiotape, transcribed verbatim and all phrases were systematically coded. Common themes and codes were collected and analysed by two assessors (MM, AAS) using directed and summative qualitative content analysis [36] methods. The 60 interviews were analysed simultaneously; as saturation had been reached no new interviews were performed.

Five steps were followed: (I) Transcripts were read to gain a contextualized impression of the discussion, and preliminary themes chosen. (ii) The data were collected without any preconceived ideas or theories. Units of meaning were identified and coded. Thus the grounded theory was applied for these analyses. (iii) Similar codes were condensed in categories and this process was repeated with each transcript. (iv) Descriptions were then summarized to establish concepts. (v) Descriptions and concepts were reviewed by the other co-author (LG) which led to the development of overarching themes or topic areas that seek to represent a cohesive interpretation of participants’ experience of fatigue.

## Results

### Patient characteristics

The sample consisted of 60 patients, 20 patients in each disease group (Table 2). The RA patients comprised 16 females and four males with a median age of 40 years, and disease duration from 1 to 20 years, with a median duration of 5 years. The AxSpA group comprised mainly males (90 %) with a median age of 34 years and median disease duration of 7 years. Finally, the fibromyalgia patients were mainly females (80 %), with a median age of 35 years and a median disease duration of 3 years (Table 2). Disease activity was in the moderate to high range (Table 2).

**Table 2:** Demographic data of the participants in the qualitative study

Characteristic	Rheumatoid arthritis	AxSpA	Fibromyalgia
Number	20	20	20
Age, years, median (range)	40 (32–60)	34 (29–55)	35 (27–54)
Female, (n) %	(16) 80 %	(2) 10 %	(16) 80 %
Disease duration, years, median (range)	5 (1–20)	7 (3–20)	3 (2–7)
Specific disease activity scale	DAS28: 3.4 (1.1–5.5)	BASDAI: 5 (2–9)	FIQ: 75 (50–90)
Patient assessment of fatigue, 0–10 NRS, median (range)	5 (2–9)	4 (1–8)	7 (3–10)

*AxSpA* Axial Spondyloarthropathy, *RA* Rheumatoid Arthritis, *DAS28* Disease Activity Score in 28 joints, *BASDAI* Bath Ankylosing Spondylitis Disease Activity Index, *FIQ* Fibromyalgia Impact Questionnaire, *NRS* Numeric rating Scale

### Main findings regarding fatigue (Table 3)

**Table 3:** Main findings regarding fatigue - common themes among different groups

Item	Common themes	Groups	Quotes
Description and causes of fatigue.	Overwhelming physical tiredness with consequences on difficulties to move	Common in all groups: RA, AxSpA and fibromyalgia	I feel that my body is broken and every movement is as if I was moving a mountain
	The need to rest more	RA and fibromyalgia	In the morning when I see my kids, I already feel tired and I even feel that I did not want to wake up
	A sense of freezing of the body	AxSpA	When I have fatigue, my body is wooden and I move with difficulty
	A toxin in the body	RA and AxSpA	It is toxic in my body, everything becomes difficult
Fatigue and pain	Close association between fatigue and pain	Common in all groups: RA, AxSpA and fibromyalgia	
Daily patterns of fatigue	Fatigue is more in the morning	AxSpA	When I get up I feel that I am very tired and I wait for one hour to be able to move
	Fatigue is more in the evening	RA, AxSpA	At the end of the day when I have finished my tasks I feel that my body is broken
	No special daily patterns	Fibromyalgia	
Consequences of fatigue	Fatigue affected physical activity to the extent that participants did their work only minimally	Common in all groups: RA, AxSpA and fibromyalgia	I do important things only
	Social activities became limited to the close family and to very important occasions.	RA and fibromyalgia (female only)	I go to weddings and condolence gatherings only, because of my fatigue
	Fatigue affected leisure activities markedly	RA and fibromyalgia	I forget what leisure means
	A difficulty in reaching the place of work	Common in all groups: RA, AxSpA and fibromyalgia	I can hardly walk to my work though it is only about 30 min
	Women told their husbands to marry again	RA and fibromyalgia	I asked my husband to marry again, he needs a healthy woman and I am too tired
	A decrease in male sexual activity	AxSpA	I get fatigued easily and this is a problem for my sexual life
Coping strategies	Dividing big tasks into smaller multiple tasks	Common in all groups: RA, AxSpA and fibromyalgia	
	Getting help from family members	RA	My mother and sisters help me to complete my daily tasks

*AxSpA* Axial Spondyloarthropathy, *RA* Rheumatoid Arthritis

#### Description and causes of fatigue

In all disease groups, fatigue was well-understood by patients. The participants described their feeling of fatigue by referring to a concept of overwhelming physical tiredness with consequences on difficulties to move.

*RA patient 5, female, 45 years: I feel that I cannot make any movement; I need help for minor movements.*

*RA patient 9, female, 32 years: Fatigue paralyzes me.*

*AxSpA patient 11, male, 51 years: I feel that my body is broken and every movement is as if I was moving a mountain.*

The need to rest more was common for patients with RA and patients with fibromyalgia, but less reported by AxSpA patients.

*Fibromyalgia patient 5, female,41 years : In the morning when I see my kids, I already feel tired and I even feel that I did not want to wake up.*

*RA patient 13, female, 22 years: Fatigue just sticks me to the bed.*

A theme that was unique to patients with AxSpA was a description of fatigue as a sense of freezing of the body.

*AxSpA patient 12, male, 23 years: When I have fatigue, my body is wooden and I move with difficulty.*

Description of fatigue as a toxin in the body was reported by one patient in AxSpA group: *AxSpA patient 4, male, 37 years: I feel that I have a toxin in my body that makes me easily tired.*

Also the same description was reported by another patient in the RA group.

*RA patient 18, female, 29 years: It is toxic in my body, then everything becomes difficult.*

Although fatigue was mainly physical, some RA and fibromyalgia patients described a link with mental fatigue/loss of cognitive functions; this was not however reported by AxSpA patients.

*RA patient 3, female, 32 years: Sometimes I feel that that I lose my concentration when I have fatigue.*

### Fatigue and pain

There was a close association between fatigue and pain among patients in the three disease groups with a general consideration that fatigue is a result of pain. For instance, when asked about fatigue, many participants respond by talking about their pain and when asked whether their answers were related to pain or fatigue, the common response was that the answers were related to both.

*Fibromyalgia patient* 4, female, 30 years old: At the end of the day I feel that all my body is suffering from severe pain.

### Daily patterns of fatigue

Patients with AxSpA had a major concern about fatigue in the morning whereas patients with RA and AxSpA both, suffered from fatigue in the evening. Patients with fibromyalgia described permanent fatigue and were not concerned with any special daily patterns.

*RA patient 16, male: at the end of the day when I have finished my tasks I feel that my body is broken.*

AxSpA *patient 9, male, 39 years: When I get up I feel that I am very tired and I wait for one hour to be able to move.*

### Consequences of fatigue

In all groups, the participants stated that fatigue influenced and affected physical activity to the extent that they did their work only within minimal limits and they did ‘important things only’.

Fatigue had a major impact on social activities of patients with RA and fibromyalgia. Social activities became limited to the close family and to very important occasions.

*RA patient 14, female, 47 years: I go to weddings and condolence gatherings only, because of my fatigue.*

Social activities seemed to be not so largely affected among patients with AxSpA. Of note that the only female with AxSpA did however report limitations of social activity similar to the limitations noticed in the other two disease groups. On the other hand males with RA or fibromyalgia were less concerned than females about alterations of social activities due to fatigue.

Fatigue also affected leisure activities markedly more in RA and fibromyalgia patients, whereas patients with AxSpA reported fatigue altered but did not render impossible, leisure activities.

*RA patient 3, male, 31 years: I forget what leisure means.*

*Fibromyalgia patient 12, female, 39 years: My health is not good enough to have leisure activities.*

*AxSpA patient 10, male, 34 years: Even when I’m tired, I just go to the café regularly.*

For patients who worked, a common theme was that they had difficulty in reaching the place of work.

*Fibromyalgia patient 13, female, 32 years: I can hardly walk to my work though it is only about 30 min.*

Fatigue affected sexual activity markedly in all groups and it was a major concern of most of the participants. A common theme that was noted for several female participants with RA and fibromyalgia was that they told their husbands to marry again as they felt that because of their fatigue, they could not satisfy their husbands fully. In AxSpA a common theme among male patients was that their sexual activity was decreased and that they became fatigued rapidly.

### Coping strategies

A common coping strategy in all disease groups was dividing big tasks into smaller multiple tasks. Another strategy noted by patients with RA was getting help from family members.

*RA patient 12, female,34 years: My mother and sisters help me to complete my daily tasks.*

Patients with fibromyalgia felt their fatigue was ignored by their families so they did not get enough help from their relatives.

## Discussion

To the best of our knowledge, this is the first qualitative study exploring the experience of fatigue among non-Western culture patients with rheumatic diseases (RA, fibromyalgia and AxSpA). This study demonstrated common experiences of fatigue, as well as aspects singular to this population, which should be taken into account when physicians and researchers assess fatigue in different countries and cultures.

The patients in all disease groups tended to describe fatigue with the same concept of overwhelming tiredness and exhaustion, with clear consequences experienced as limitations of activities of daily living which was consistent with previous qualitative studies in Western cultures [37, 38]. However for the patients interviewed in the current study, fatigue was mainly related to physical impact of disease and more rarely to mental impact. In other qualitative studies of fatigue, fatigue associated with RMDs appeared to have as much mental impact (with descriptions of loss of energy and cognitive impairment) as physical (with descriptions of inability to do daily activities). Thus, this study suggested that fatigue is perceived by Egyptian patients with different RMDs as a specific entity, but the fatigue experiences related much more strongly to physical aspects for these patients.

A unique description of fatigue in the present study that we did not find in previous qualitative studies was that fatigue for some patients was a sense of a ‘toxin’ passing through their bodies. This may give the idea that for these patients, fatigue is a new (and maybe a temporary) unusual tiredness. This concept of considering a disease (RA and systemic lupus erythematous) and the pharmacological treatments for these diseases as toxins, was also perceived by South-Asian patients living in the UK [22]. However, in the present study treatments and their perception was not explored.

Another culture specific aspect of our patients’ experience of fatigue was the response to the impact of fatigue on sexual function. This impact of fatigue was important and easily expressed by patients in the present study. As in previous studies [39–41], patients of both genders felt that fatigue was a major obstacle that limited their sexual activities, thus this issue appears to not be culture-specific. However the solutions proposed may be culture-specific since several women had asked their husbands to marry again (which is a possibility in the Egyptian and Islamic culture) because of the impact of their fatigue on their sexual life, and they expressed that a second wife was the easiest solution or coping strategy for this consequence of their fatigue.

Limitations of social activities due to fatigue could be also detected as specific to the Egyptian and Islamic culture of our group of patients. We noticed that limitations of social activities were more severe in women than men. This gender-specific difference was also briefly alluded to in previous qualitative studies from European countries [42, 43] but appeared more marked in the present study as it was noticed in almost all the interviewed patients. This difference may be because the participation of women in social gatherings in the Egyptian culture is not limited to attendance (like what is expected for men); many physically demanding duties are expected from a woman at a social gathering, especially at wedding parties and condolence gatherings which were a major concern for our female patients.

On the other hand, many other aspects of our patients’ experience of fatigue were identical to that presented in previous studies of patients from different cultures. One of these aspects is the relation between pain and fatigue. As noticed in previous qualitative studies there was a large overlap between the patients’ perception of fatigue and pain [37, 44]. This indicates that it may be difficult for patients to fully separate different aspects of disease impact, and perhaps even more so in the Egyptian culture where patients are not habitually describing their symptoms regarding quality of life impairment to their physicians, in the context of usual care. However, the overlap between different symptoms has also been noted in other RMD studies in other cultures [30].

The coping strategies described by patients to overcome their sense of fatigue or its consequences, did not appear to have cultural specificities when compared to previous studies [43, 45, 46]. Although the family structure is culturally important in Egypt [47], obtaining help from close family was not always easy for some patients particularly the fibromyalgia patients perhaps because of the lack of joint deformity or other obvious sign of disease. Social support is very important for coping strategies; the relationship between RMDs and social support across diverse cultures should be investigated further.

The current study showed some disease specific differences in patients’ views, based on the three patient groups. Patients with AxSpA described their feeling of fatigue as a sense of freezing of the body which may be attributed to the specific nature of AxSpA in which stiffness is a major part of the disease. This appears consistent with the descriptions of fatigue in patients with axSpA from the United Kingdom [30]. Patients with AxSpA suffered from fatigue mainly in the mornings; this may confirm previous studies [25, 48] which showed that the degree of fatigue in AxSpA is related to the degree of morning stiffness.

Finally disease-specific differences were found in the effect of fatigue on leisure activities. While patients with RA or fibromyalgia felt that leisure activities were almost impossible, patients with AxSpA were able to maintain these activities somewhat more easily. This confirms previous findings and thus does not appear to be specific to the Egyptian and Muslim culture of our patients [45, 46].

Although this study is limited by the small sample size and the potential lack of data saturation, rigorous data interpretation methods were applied and comparisons were performed with the literature. Thus, this qualitative study allowed a first exploration of Egyptian Muslim patients’ experiences of fatigue, and we believe some important insights have been gained. Generalization of the results of this study should be considered with caution as in all qualitative studies.

In conclusion this study unveiled some aspects of our patients’ experience of fatigue which may be specific to the Egyptian and Muslim culture such as the description of fatigue as a more physical rather than mental impact of the disease, the response to the effect of fatigue on sexual function and the gender specific (women more than men) limitation of social activities. Other aspects of patients’ experience of fatigue such as overlap between the patients’ perception of fatigue and pain and coping strategies were similar to the findings in previous studies. Future studies should explore further culture-specific aspects of fatigue.
